# Residual Block Based Nested U-Type Architecture for Multi-Modal Brain Tumor Image Segmentation

**DOI:** 10.3389/fnins.2022.832824

**Published:** 2022-03-09

**Authors:** Sirui Chen, Shengjie Zhao, Quan Lan

**Affiliations:** ^1^School of Software Engineering, Tongji University, Shanghai, China; ^2^Department of Neurology, First Affiliated Hospital of Xiamen University, Xiamen, China

**Keywords:** brain tumor, multi-modal image segmentation, MRI, CNN, ResNet, UNet

## Abstract

Multi-modal magnetic resonance imaging (MRI) segmentation of brain tumors is a hot topic in brain tumor processing research in recent years, which can make full use of the feature information of different modalities in MRI images, so that tumors can be segmented more effectively. In this article, convolutional neural networks (CNN) is used as a tool to improve the efficiency and effectiveness of segmentation. Based on this, Dense-ResUNet, a multi-modal MRI image segmentation model for brain tumors is created. The Dense-ResUNet consists of a series of nested dense convolutional blocks and a U-Net shaped model with residual connections. The nested dense convolutional blocks can bridge the semantic disparity between the feature maps of the encoder and decoder before fusion and make full use of different levels of features. The residual blocks and skip connection can extract pixel information from the image and skip the link to solve the traditional deep traditional CNN network problem. The experiment results show that our Dense-ResUNet can effectively help to extract the brain tumor and has great clinical research and application value.

## 1. Introduction

Tumors are localized cell growths in the body that constitute a tumor for various reasons. There are two classifications of tumors, benign and malignant. Cancer is usually a malignant tumor, and is a common malignant diseases that threaten human health. Therefore, accurate segmentation and subsequent quantitative analysis of tumor images is a routine and critical step in treatment.

MRI is based on the principle that the electromagnetic signal released by the instrument has different attenuation in different structures in the body to obtain the electromagnetic signal from the body and reconstruct the body information (Vaughan et al., 2006). Compared with other traditional medical imaging techniques such as X-ray and CT imaging, MRI can provide early detection of smaller and more microscopic lesions (Nandpuru et al., 2014). Due to the complexity of human organ tissues, the same imaging technique usually results in images of different modalities, where different modalities also reveal different pathological information (Legg et al., 2015). MRI provides four different imaging sequences obtained by several different imaging display techniques, and these four different imaging sequences can provide complementary information for clinical diagnosis.

**Figure 1:**
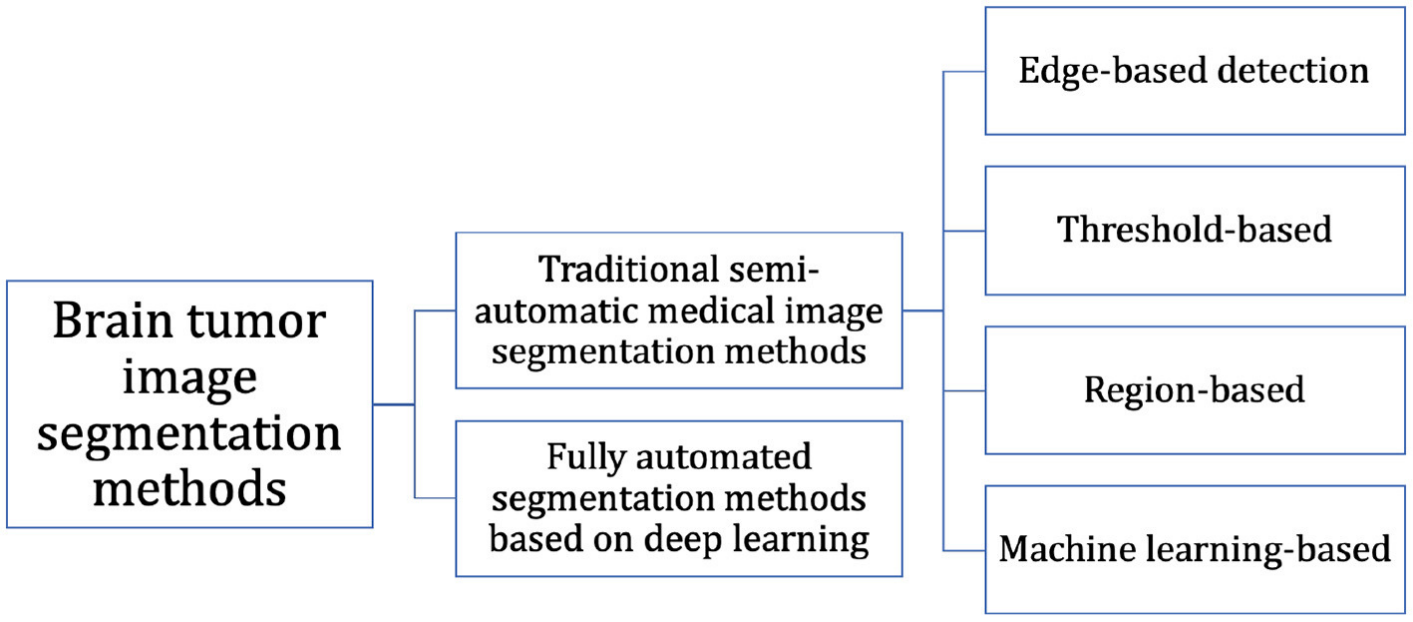
Classification of brain tumor image segmentation methods.

At present, the segmentation of MRI brain tumor images is mainly based on the experience of expert doctors, but it is difficult to accurately segment MRI brain tumor images preoperatively because of the time-consuming and repetitive work, and the subjective judgment of the physician can interfere with it. In recent years, many researchers have improved natural image segmentation algorithms and then migrated them to brain tumor segmentation tasks, resulting in a large number of effective research methods and findings. As shown in Figure 1, we can classify them into two categories: (i) Fully automated segmentation methods based on deep learning and (ii) Traditional semi-automatic medical image segmentation methods.

**Edge-based detection**: The basic idea of the edge detection-based image segmentation method is to first find its edge pixels in the image and then aggregate these pixels to form the desired region edges and segment the image with the region edges. Aslam et al. (2015) proposed an improved edge detection algorithm for brain tumor segmentation based on the operator (Sobel, 1970) of the first-order derivatives, which combined the Sobel method and the image dependent thresholding method and used a closed contour algorithm to find the different regions. Finally, the intensity information within the closed contours was used to extract the tumor from the image.

**Threshold-based**: The idea of the algorithm is that first, the grayscale features of the image are computed to obtain one or more grayscale thresholds, and then the grayscale values of each pixel in the image are compared with each other and with the computed thresholds. Finally, based on the comparison results, the pixels can be classified into different classes. Kittler and Illingworth (1986) proposed a computationally efficient solution to the minimum error thresholding problem applicable to multiple threshold selection. Saad et al. (2010) used the histogram thresholding technique in segmentation to detect pixels with high or low intensity.

**Region-based**: Region-based image segmentation algorithms can be divided into two basic forms: (i) The global departure form, where segmentation is performed gradually until the desired region and (ii) The region growth form, where individual image pixels are started and gradually merged, which in turn forms the segmented region we need. Kaus et al. (2001) used a region-growing approach to segment MRI brain tumor images based on signal intensity values.

Since the main idea of traditional segmentation algorithms is to start from features such as texture and grayscale values between different tissues in medical images, the main challenges of this type of algorithms are the large grayscale similarity and uneven distribution between brain tissues in MR images, while the grayscale values contain too little information and the variability between different cases, which can affect the final segmentation accuracy.

**Machine learning-based**: Machine learning-based image segmentation algorithms require training a model from a certain number of images with well-defined mapping relationships between image features and labels to learn the segmentation laws. Weakly supervised learning methods and semi-supervised learning methods were first developed, mainly random forests (Shi and Horvath, 2005), Adaboost (Rtsch et al., 2001), K-Means clustering (Hartigan and Wong, 2013), support vector machines (SVMs) (Zhang et al., 2006), etc. The method proposed by Abdelmaksoud et al. (2015) can not only take advantage of the fuzzy C-means algorithm to obtain high accuracy, but also make full use of K-Means clustering to obtain the minimum computation time, which decreased the segmentation time while improving the segmentation accuracy. Tustison et al. (2014) combined the random forest model with regularized probability and used the probability map generated by this model for Markov random field, which finally obtained better results for brain tumor segmentation. Mahmood and Basit (2016) proposed an automatic segmentation framework for ischemic stroke lesion segmentation in multi-spectral MRI based on random forest. However, the problem with the machine learning-based approach is that the selection and labeling of image features require the use of specialized medical knowledge, which limits the development of such methods.

CNNs were originally used in areas such as handwritten alphabet classification, but the input of such images was already fixed in size, so when migrating such networks to the field of image segmentation, it also started by classifying blocks of images of fixed size. Moeskops et al. (2016) came up with a method to automatically segment MRI brain images. Their network obtained multi-scale information for each voxel, using multiple convolutional kernels of different sizes. The method did not rely on explicit features but learns to identify important information about the classification based on the training data. However, this method was used only for a single modality MRI image. Hou et al. (2016) proposed a segmentation network that cut a complete image into fixed-size image blocks, after which they were convolved separately to extract features and finally classified for the central pixel points to obtain the final segmentation results. Their method focused the classification from the image level to the pixel level. Brosch (2015) proposed a 3D deep neural network, this model had both convolutional and deconvolutional layers and combined feature extraction and segmentation prediction, this method has high accuracy but was too computationally intensive.

The high cost of acquiring medical images and the relatively small dataset for medical image segmentation also pose a problem for deep learning that requires large amounts of data. And due to the layer-by-layer convolution of neural networks, the accuracy of segmentation is reduced while increasing the computational effort. Multi-modal MRI brain tumor image segmentation can make full use of the feature information of different modalities in MRI thus improve the effectiveness of segmentation, which is one of the recent research hotspots in the field of brain tumor image processing. Using artificial intelligence technology, a fully automated method for brain tumor image segmentation can be designed so that it can support physicians' analysis and diagnosis, which is the best way to solve the above problem.

In general, this article focuses attention on how to fully and efficiently utilize multi-modal MRI image information, proposes a multi-modal nested dense ResU-Net segmentation network, and validates the effectiveness of the algorithm on an open-source dataset of brain tumors.

## 2. Materials and Methods

**Figure 2:**
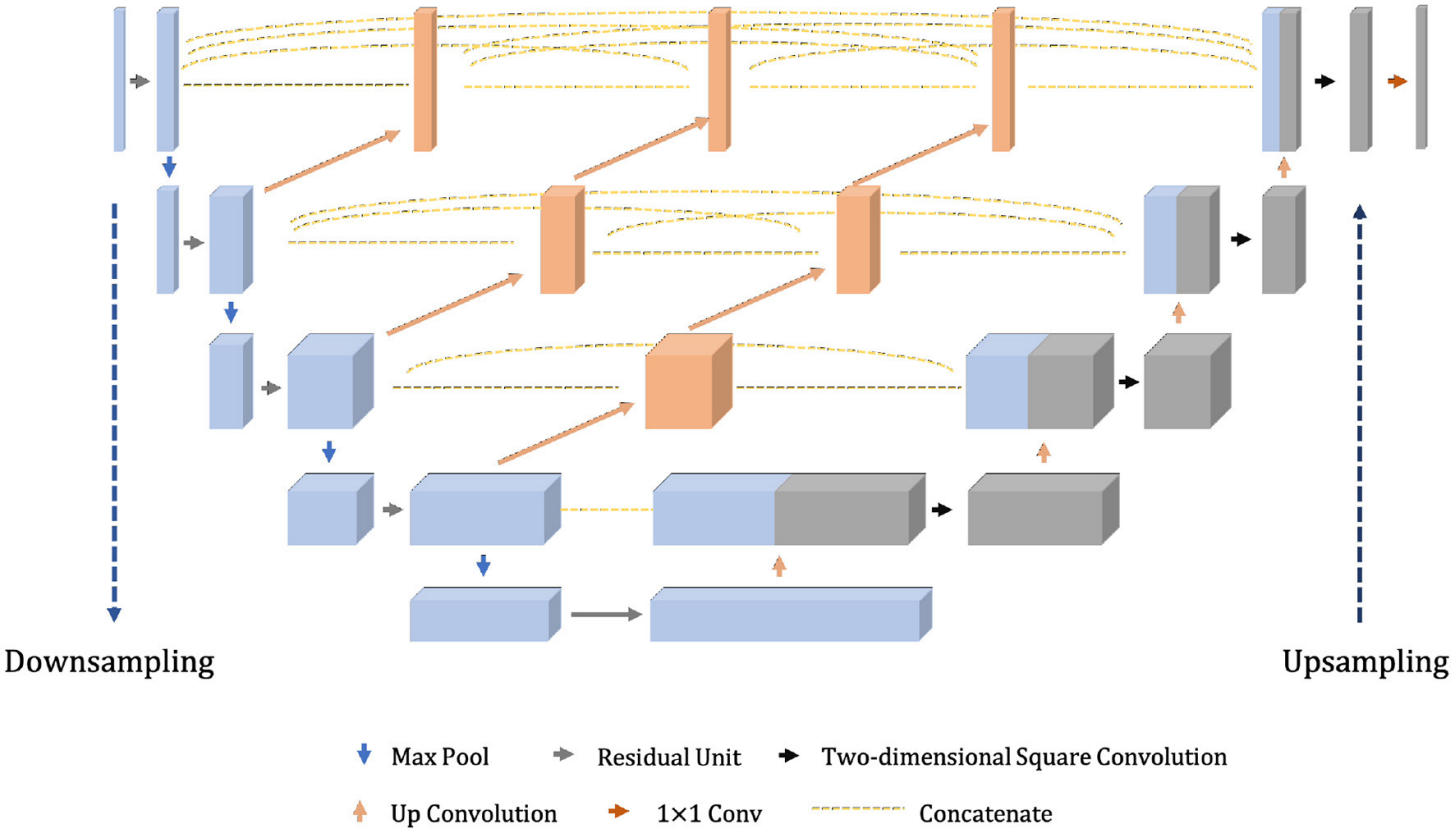
The overall structure of our proposed Dense-ResUNet.

The model proposed in this section, given the abbreviation Dense-ResUNet, is a multi-modal MRI brain tumor segmentation model based on the traditional U-Net (Ronneberger et al., 2015) and U-Net++ (Zhou et al., 2018), and the network structure is shown in Figure 2. The left is the downsampling path, which effectively extracts the image features through a series of successive convolution and downsampling operations, and reduces the image size while increasing the number of channels; the right is the upsampling path, which successfully recovers the image size, improves the image segmentation accuracy and allows better reconstruction of details through a series of successive transposition convolution operations; the middle is a series of nested dense convolutional blocks. The structure bridges the semantic disparity between the feature maps of the encoder and decoder before fusion. Based on this modular design, our model can train the network by adding a small number of convolutional layers to extract good medical image features.

**Figure 3:**
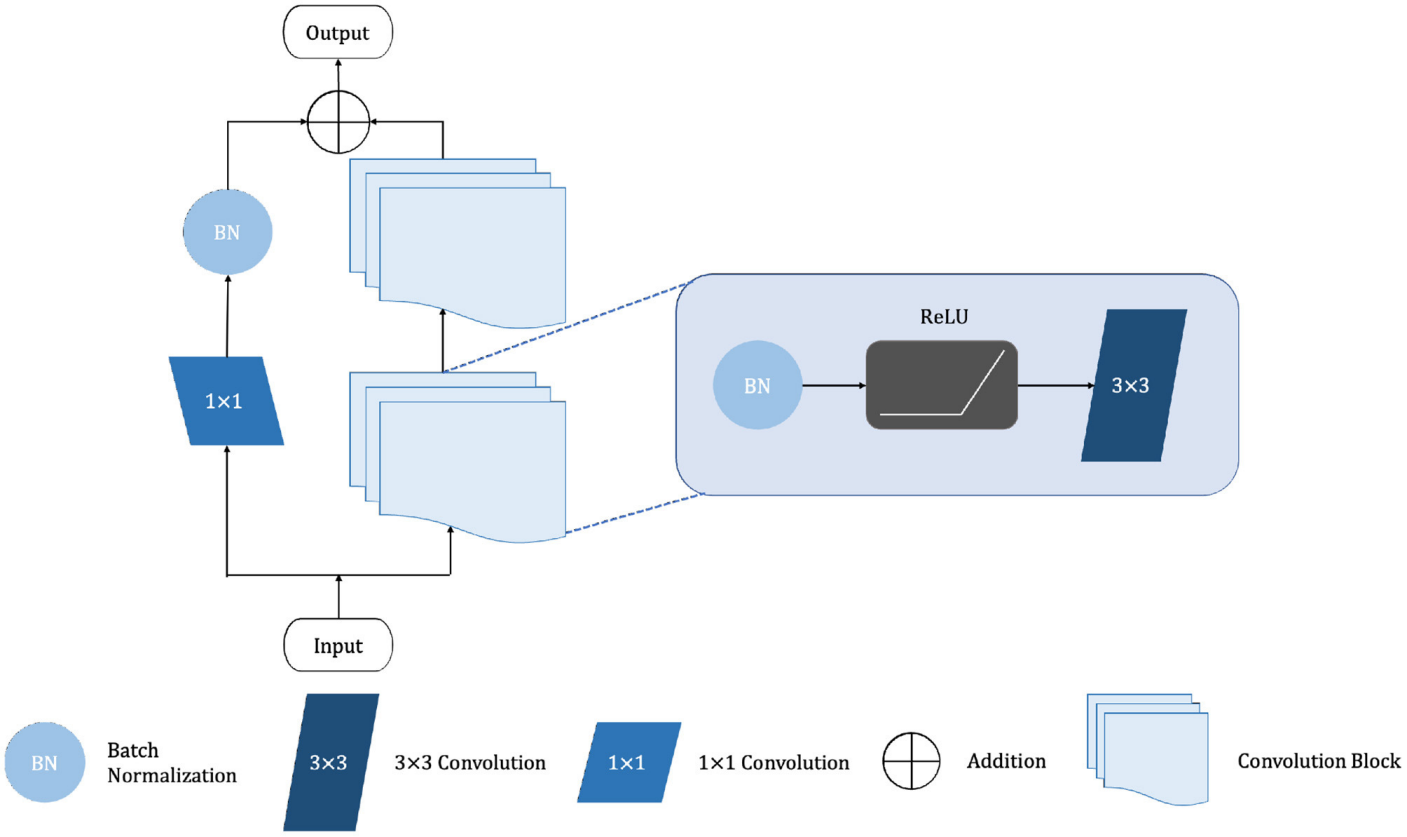
The framework of residual unit.

The shalllow convolution structure cannot fully capture the complex structure of the image, but the deep convolution and redundant structure of the stack lead to the gradient vanishing and tearing problem. Therefore, we use a residual unit to extract pixel information from the image and skip the link to solve the traditional deep CNN network problem. The structure of the residual unit is shown in Figure 3 and the specific embedding method is shown in **Figure 5**. Each layer in the constructed Dense-ResUNet model needs to use convolutional blocks so that feature extraction can be performed. Each of these convolutional blocks consists of 2 convolutional units. In each of these 2 convolutional units, a BatchNorm (BN) layer is included to improve model convergence. The activation is then performed with the ReLU function, which is used to improve the non-linearity of the function.

### 2.1. ResNet Architecture Overview

Each of the convolutional layers contains feature maps. When a pixel in the image is scanned by the convolution kernel and makes full use of the complex content information in the environment to generate semantic image features, the image features with content and space are expressed by the activation function (ReLU) as Equation (1):


(1) $$\begin{array}{l} X_{j}^{l+1}=f\left(t_{i}^{l+1}+\underset{i\in I_{j}}{\sum }X_{i}^{l}⋇k_{ij}^{l+1}\right) \end{array}$$

Where ${X_{j}}^{l+1}$ represents the feature map after the (*l* + 1) − *th* layer, *t* represents the offset term, ${X_{i}}^{l}$ represents the input feature in the (*l* + 1) − *th* layer, *f* is the activation function (rectifier linear unit, ReLU), *I*_*j*_ represents a series of input eigenmatrices, ⋇ represents the convolution operation, *k* represents the convolution kernel.

By reducing the dimension of the image features, the pooling layer can represent the high-level content and semantics:


(2) $$\begin{array}{l} X_{j}^{l+1}=t_{j}^{l+1}+X_{j}^{l}⊛k_{j}^{l+1} \end{array}$$

Where ⊛ refers to pooling operations in the convolutional structure. Finally, the fully connected layer takes the maximum likelihood function as the prediction layer to carry out the image classification task.

**Figure 4:**
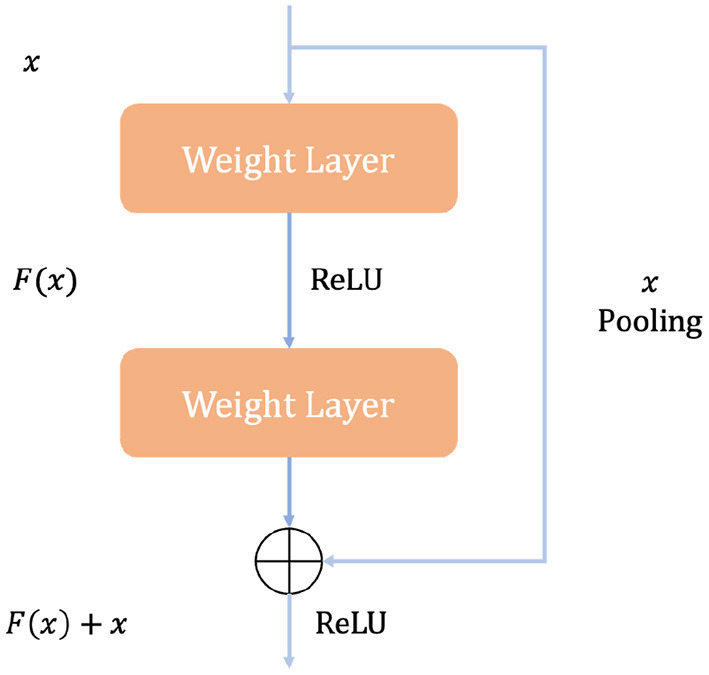
The basic block of ResNet.

Figure 4 illustrates how a building block works in ResNet. A quick connection mechanism between each initial input *X* and the module output *H*(*X*) is introduced, so that the input can learn the residual expression *F*(*X*) = *H*(*X*) − *x* directly to model the target output [*F*(*X*) + *X*]. With such a mechanism, the precision problem and performance degradation due to too many stacked convolution structures can be avoided, and such a quick connection mechanism can perform identity mapping in multi-layer structures. It serves as a reference for the input elements in each layer and learns to form the corresponding residual function instead of some function blocks with no practical value [*F*′(*X*)]. It is easier to optimize the propagation using such a mapping method and thus can significantly increase the number of layers in the network structure. The formula for the residual mapping function is as follows:


(3) $$\begin{array}{l} F=W_{2}f\left(W_{1}x\right) \end{array}$$

(4) $$\begin{array}{l} y=F\left(x,W_{i}\right)+W_{s}x \end{array}$$

Where *f*(·) represents the ReLu activation function and then generates the corresponding output *y* through a Shortcut Connection and the second ReLU function. When we switch the dimensions of the input and output representations, we can use *W*_*s*_ for linear transformation.

### 2.2. UNet Architecture Overview

**Figure 5:**
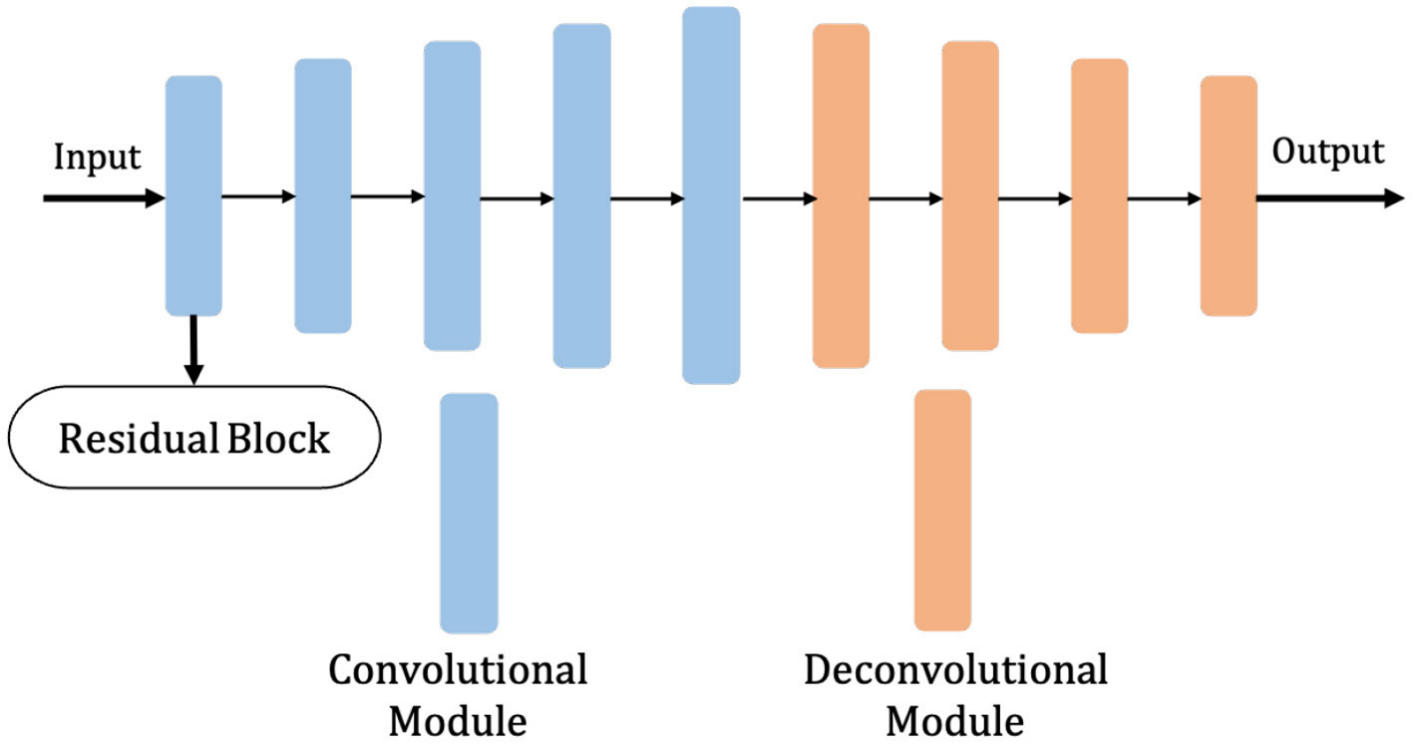
Dense-ResUNet model framework diagram.

Medical images usually have small samples and most medical images have complex modalities, and the U-Net model proposed by Ronneberger et al. (2015) has an excellent performance in processing medical images with small samples due to its excellent learning ability. The main idea of U-Net is to connect a network with a similar structure to the previous layer behind the downsampling stage, and then recover the resolution of the image output by applying upsampling and combining the output of upsampling with downsampling having high-resolution features combined in these ways to better extract the edge features of the image. Figure 5 demonstrates the network framework.

Suppose *F*_*mn*_ denotes the convolution operation performed at position (*m, n*), the size of the two-dimensional convolution kernel is *a* × *b*, and the two-dimensional convolution is calculated as Equation (5).


(5) $$\begin{array}{l} F_{mn}=f\left(s_{mn}+\overset{a-1}{\underset{i=0}{\sum }}\overset{b-1}{\underset{j=0}{\sum }}w_{ij}\cdot X_{\left(m+i\right)\left(n+j\right)}\right) \end{array}$$

Where *s* refers to stride, *w* refers to convolution kernel and *X* is the input.

From Figure 2, we can see that the feature maps of the encoder go through a dense convolution block. The convolution layers' number of the blocks depends on the pyramid level. We assume that $X^{l_{d},l_{c}}$ is a node in the model, where *l*_*d*_ refers to the downsampling layer along the encoder and *l*_*c*_ refers to the convolution layer of the dense block along the skip connection. Meanwhile, we define $x^{l_{d},l_{c}}$ as the output of $X^{l_{d},l_{c}}$, then the $x^{l_{d},l_{c}}$ can represent the feature maps as Equation (6).


(6) $$\begin{array}{l} x^{l_{d},l_{c}}=\{\begin{array}{ll} \Delta \left(x^{l_{d}-1,l_{c}}\right), & where\;l_{c}=0\\ \Delta \left(\left[{\left[x^{l_{d},l_{k}}\right]}_{l_{k}=0}^{l_{c}-1},F\left(x^{l_{d}+1,l_{c}-1}\right)\right]\right), & where\;l_{c}>0 \end{array} \end{array}$$

Where Δ(·) refers to the convolution operation followed by ReLU, F(·) refers to the upsampling operation and [] refers to the concatenate operation.

The role of the pooling layer is to perform merge operations on the input data. We conducted the maximum pooling approach in this article. We calculate the height and weight of the output according to Equations (7) and (8).


(7) $$\begin{array}{l} H_{out}=\left\lfloor \frac{H_{in}+2\times p_{i}-d_{i}\times \left(k_{i}-1\right)-1}{s_{i}}+1\right\rfloor \end{array}$$

(8) $$\begin{array}{l} W_{out}=\left\lfloor \frac{W_{in}+2\times p_{j}-d_{j}\times \left(k_{j}-1\right)-1}{s_{j}}+1\right\rfloor \end{array}$$

Where *p* refers to the padding, *d* refers to the dilation, *H* refers to height and *W* refers to weight.

The objective function used in the network proposed in this article is dice loss, which is a concept first proposed by Milletari et al. (2016). Its functional expression is shown in Equation (9) and the function takes values in the range [0,1].


(9) $$\begin{array}{l} D=\frac{2\sum_{i}^{N}p_{i}g_{i}}{\sum_{i}^{N}p_{i}^{2}+\sum_{i}^{N}g_{i}^{2}} \end{array}$$

where *p*_*i*_ is the pixel value of point *i* in the prediction result and *g*_*i*_ is the pixel value of point *i* in the label, resulting in the gradient equation as in Equation (10).


(10) $$\begin{array}{l} \frac{\partial D}{\partial p_{i}}=2\left[\frac{g_{i}\sum_{i}^{N}p_{i}^{2}+\sum_{i}^{N}g_{i}^{2}-2p_{i}\sum_{i}^{N}p_{i}g_{i}}{{\left(\sum_{i}^{N}p_{i}^{2}+\sum_{i}^{N}g_{i}^{2}\right)}^{2}}\right] \end{array}$$

### 2.3. Multi-Modal MRI

**Figure 6:**
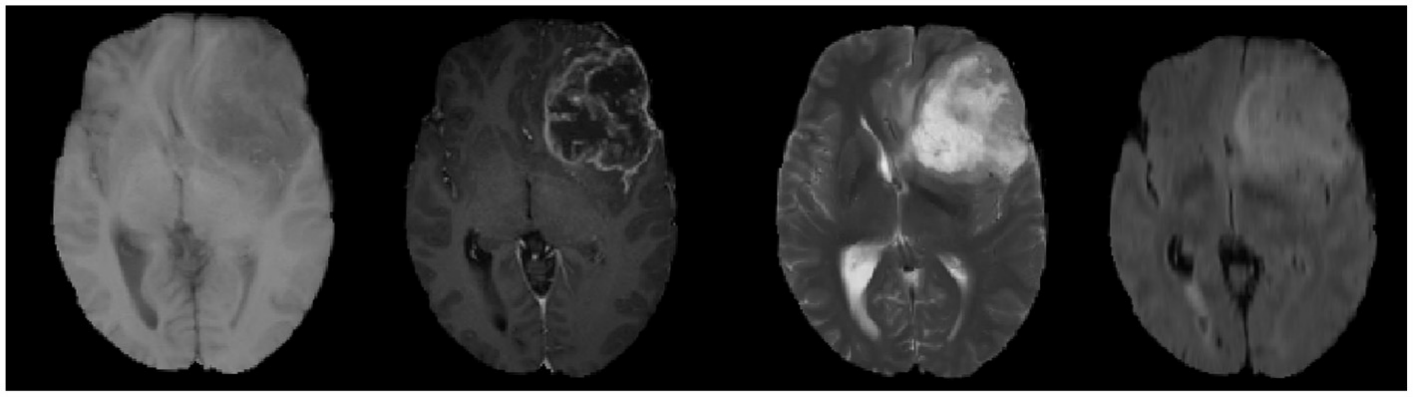
MRI images of four different modalities.

MRI is performed by varying the direction and intensity of the magnetic field thus obtaining different imaging sequences, which is called multi-modal. Four main imaging forms of multi-modal usually exist, namely: T1-weighted form, T1c-weighted form, T2-weighted form, and FLAIR-weighted form. The four modalities are shown in order from left to right in Figure 6, and these pictures also show that the brain tumor image has complex texture and obvious structural information. The imaging of different modalities can highlight different characteristic information of the tumor. By deeply analyzing and thinking about multi-modal images, we can obtain more comprehensive information of brain tumor images and provide help to the research related to brain tumor lesion regions.

### 2.4. Datasets

We use BraTS 2018 dataset to train the modal, and it consists of high-grade gliomas (HGG), low-grade gliomas (LGG), and real known tumor segments repeatedly hand-drawn by several medical experts. Four MRI sequences were performed in each case. All imaging sequences of the same subject were preprocessed and stripped from the skull, and all 3D MRI images were volume sized 240 × 240 × 155 and they were aligned, interpolated, and rescaled to achieve the same resolution (1*mm*^3^). A total of five types of intra-tumor structures were available in the dataset: (i) normal tissue; (ii) edematous regions (Peritumoral Edema, ED); (iii) necrosis (NCR); (iv) Non-enhancing Tumor (NET); and (v) Enhancing Tumor (ET).

### 2.5. Multi-Modal Fusion of Brain Tumor Images

Multi-modal brain tumor image fusion is the synthesis of multiple images into a new image. The fusion result is more beneficial to human recognition and automatic machine detection due to the ability to exploit the spatio-temporal correlation and information complementarity of multiple brain tumor images, and to make the fused image obtained a more comprehensive and clear description of the scene.

Our proposed method performs pixel-level image fusion of images and constructs multi-modal image channels, as show in Equation (11). The image obtained after pixel-level image fusion preserves the original information to the maximum extent, which is conducive to further analysis, processing, and understanding of the image, and is also able to expose potential targets, facilitating the operation of judgment to identify potential target pixel points, which is the only way to preserve as much information as possible in the source image, making the fused image increased in both content and detail, an advantage that is unique and exists only in pixel-level fusion.


(11) $$\begin{array}{l} F_{COMB}^{\left(i\right)}=F_{Flair}^{\left(i\right)}+F_{T1}^{\left(i\right)}+F_{T1c}^{\left(i\right)}+F_{T2}^{\left(i\right)} \end{array}$$

## 3. Experimental Configurations

### 3.1. Evaluation Metrics

In this article, the evaluation metrics proposed by MICCAI BraTS are used to evaluate the performance of the proposed segmentation method. The dataset MICCAI BraTS provides five types of labels for training the network. Different structures in the five types of labels are divided into three regions to meet the medical clinical applications in real situations, and the evaluation method described in this chapter also focuses on these three tumor regions.

(i) **Enhancing tumor (ET)**, which is present only in high-grade gliomas and is a characteristic enhancing core structure; (ii) **Tumor core (TC)**, a collection of three types of tumors: necrotic, non-enhancing and enhancing tumors; (iii) **Whole-area tumor (WT)**, which is a collection of all intra-tumor structures.

*T*_1_ denotes the real brain tumor area (Ground Truth). *T*_0_ denotes other parts, i.e., normal brain regions. *P*_1_ denotes the predicted brain tumor area, *P*_0_ denotes the other parts are the predicted normal brain regions. It is assumed that brain tumor is a positive sample and normal brain tissue is a negative sample. In the actual evaluation, (i) TP: the part of brain tumor correctly segmented by the model; (ii) TN: the part of normal tissue correctly segmented by the model; (iii) FP: the part of normal tissue but segmented as brain tumor; and (iv) FN: the part of brain tumor but segmented as normal tissue, which have also been shown in Table 1. Thus, we utilize the following evaluation metrics to evaluate our image classification model:

**Table 1:**
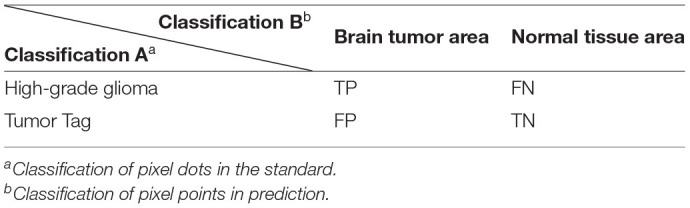
Pixel point classification labels.

(i) Dice Similarity Coefficient (DSC) is a kind of set similarity measure, usually used to calculate the similarity of two samples, the higher the Dice coefficient means that the segmentation result of the algorithm is closer to the real result.


(12) $$\begin{array}{l} Dice\left(P,T\right)=\frac{\left|P_{1}\cap T_{1}\right|}{\frac{\left(\left|P_{1}|+|T_{2}\right|\right)}{2}}=\frac{2TP}{FP+2TP+FN} \end{array}$$

(ii) Sensitivity indicates the intersection of the results of the segmentation algorithm and the real results than the value of the real results, the larger the value of this evaluation index means that the segmentation results are closer to the real results.


(13) $$\begin{array}{l} Sensitivity=\frac{\left|P_{1}\cap T_{1}\right|}{\left|T_{1}\right|}=\frac{TP}{TP+FN} \end{array}$$

(iii) Positive Predictive Value (PPV) refers to the proportion of cases that are tumors among the number of positive samples of tumor segmentation results to be evaluated.


(14) $$\begin{array}{l} PPV=\frac{TP}{TP+FP} \end{array}$$

(iv) Hausdorff distance, which represents the maximum distance between the edge points segmented by the algorithm and the actual edge points.


(15) $$\begin{array}{l} Hausdorff\left(P,T\right)=\max\left\{h\left(P,T\right),h\left(T,P\right)\right\} \end{array}$$

### 3.2. Data Pre-processing

**Figure 7:**
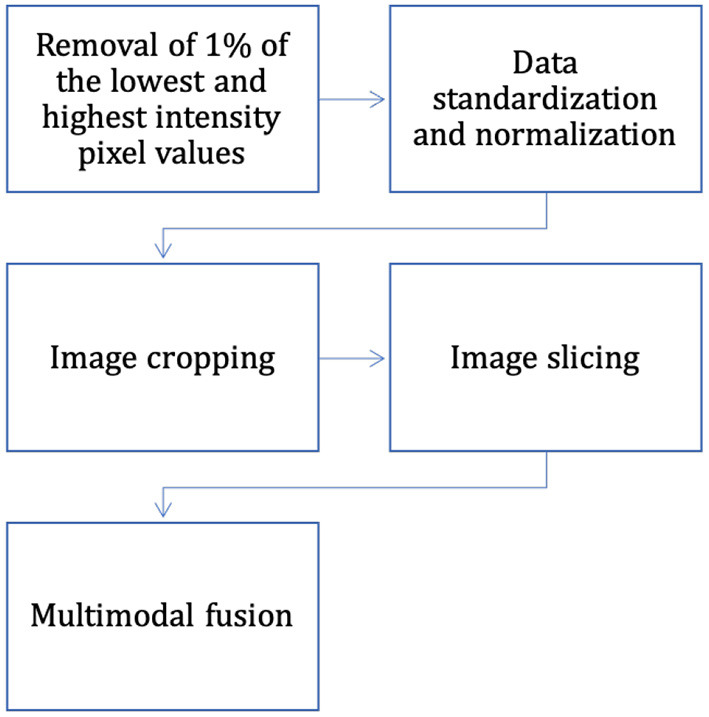
The process of data pre-processing.

The dataset has completed the basic steps of brain MRI image processing (image alignment, cranial separation, etc.), but due to the specificity of medical images, there are some differences in MRI images obtained from the same patient machine trial in the same scanner at different time points. Considering the variability of MRI brain tumor image data distribution, to make the contrast and intensity range of patient MRI images similar, this section standardizes and normalizes the pixel intensity to balance the difference of gray value between images, and the process is as follows (also shown in Figure 7):

(1) Removal of 1% of the lowest and highest intensity pixel values.

(2) For each case, the mean value of the image is subtracted and divided by the standard deviation of the image, and data normalization is performed on the data within each input channel to obtain the standardized image.


(16) $$\begin{array}{l} F=\frac{X-\mu }{std} \end{array}$$

In Equation (16), the *X* denotes the image matrix, and μ represents the pixel-average value of the image.


(17) $$\begin{array}{l} std=\max\left(\sigma ,\frac{1}{\sqrt{N}}\right) \end{array}$$

In Equation (17), the σ denotes the standard variance of the image, and *N* denotes the total number of pixels of the matrix *X* represented by the image.

(3) Normalization operation is performed for image slices to compress their pixel intensities.

The intensity of the MRI images of the brain after the three processing steps described above is in the range of [0, 1]. Most medical images are 3D data, but the expert annotations in the BraTS dataset are for 2D axial slices rather than 3D images. In this article, two-dimensional brain tumor images were used for analysis as using 2D slices as network input can also provide enough features to identify each tumor region; therefore, the only way to fit the two-dimensional network was to slice the three-dimensional data into two-dimensional data. The problem of segmenting brain tumors in MRI images is associated with a large data imbalance. To reduce the imbalance of categories, we crop the image slices from the axial plane extraction into 2D image blocks of 128 × 128. The cropped sampled image blocks not only well avoid the zero-intensity pixel blocks, but also reduce the size of normal tissue regions, which are helpful to alleviate the data imbalance problem. In addition, this also reduces the computational effort and the training time.

### 3.3. Implementation Details

The Pytorch framework is used to implement the model and the experiments are done on Ubuntu 20.04 system with an Intel Core i7 3.5GHz processor and an NVIDIA TITAN 2080Ti graphics card. Eighty percent (i.e., 233 patients) was used to train the model There are four hyperparameters set for Adam in Pytorch: lr (learning rate), smoothing constant betas, eps and weight_decay. The parameters of our proposed model are shown in Table 2.

**Table 2:** Training parameters setting.

**Parameter**	**Value**
Learning rate	0.0003
Batch_size	10
Early_stop	20
Epochs	1000
Optimizer	Adam

### 3.4. Baseline Methods

We compare our model with the following baseline methods:

**FCN32s** (Shelhamer et al., 2016): It can accept input image of arbitrary size, and use a deconvolution layer to upsample the feature map of the last convolution layer. It recovers images to the same size as the input image, which can produce a prediction for each pixel while preserving the spatial information in the original input image.

**UNet** (Ronneberger et al., 2015): The main idea is to connect a network with a similar structure to the previous layer behind the downsampling stage, and then recover the resolution of the image output by applying upsampling. Finally combine the upsampled output with the downsampled output which has high-resolution features. By these steps the edge features of the image can be better extracted.

**Attention-UNet** (Oktay et al., 2018): The attention mechanism is introduced to learn the importance of each element with respect to the target. It limits the activation to the region with segmentation and reduces the activation value of the background to optimize the result.

**ResNet-UNet**: UNet with residual block helps to solve the gradient vanishing and gradient exploding problems and train deeper networks while ensuring good performance.

### 3.5. Performance Comparison

**Figure 8:**
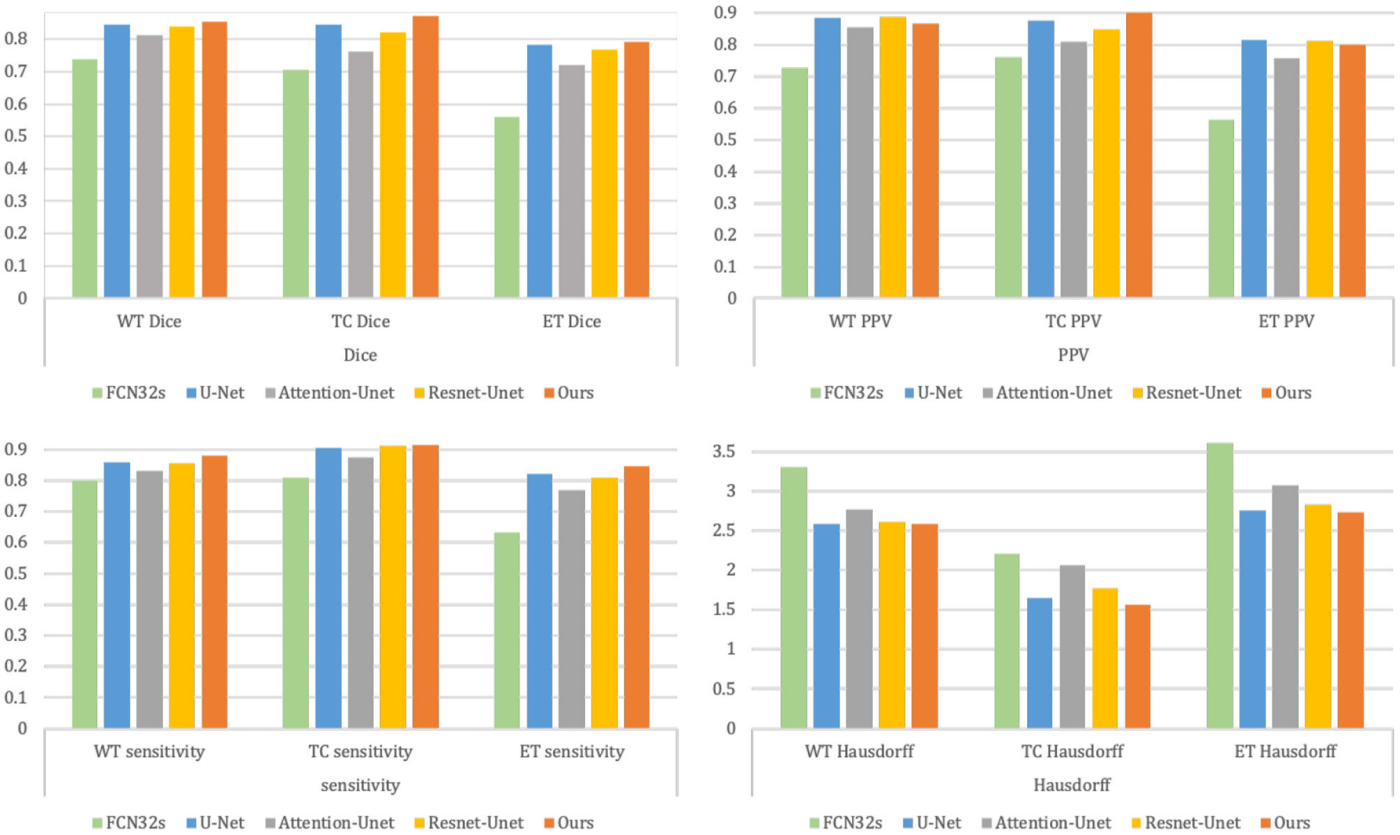
Results comparison between the baseline methods and our proposed methodology.

Table 3 and Figure 8 summarize the baseline results compared to our proposed methodology. It is apparent from Table 3 that our proposed framework outperforms the other four approaches across 9 metrics and achieves the highest Dice and Sensitivity, and the results are shown in bold values.

**Table 3:** Results comparison.

**Evaluation metrics**	**FCN32s**	**UNet**	**Attention-UNet**	**ResNet-UNet**	**Ours**
WT Dice	0.7376	0.8450	0.813	0.8384	**0.8529**
TC Dice	0.7070	0.8454	0.7633	0.8228	**0.8705**
ET Dice	0.5610	0.7817	0.7218	0.7685	**0.7908**
WT PPV	0.7287	0.8859	0.8567	0.8887	0.8686
TC PPV	0.7599	0.8775	0.8105	0.8478	**0.9050**
ET PPV	0.5634	0.8154	0.7583	0.8127	0.7991
WT Sensitivity	0.7986	0.8595	0.8296	0.8553	**0.8818**
TC Sensitivity	0.8082	0.9069	0.8739	0.9102	**0.9145**
ET Sensitivity	0.6329	0.8203	0.7698	0.8099	**0.8453**
WT Hausdorff	3.3011	2.5787	2.7640	2.6126	2.5804
TC Hausdorff	2.2013	1.6516	2.0579	1.7696	**1.5660**
ET Hausdorff	3.6041	2.7487	3.0706	2.8314	**2.7331**

**Figure 9:**
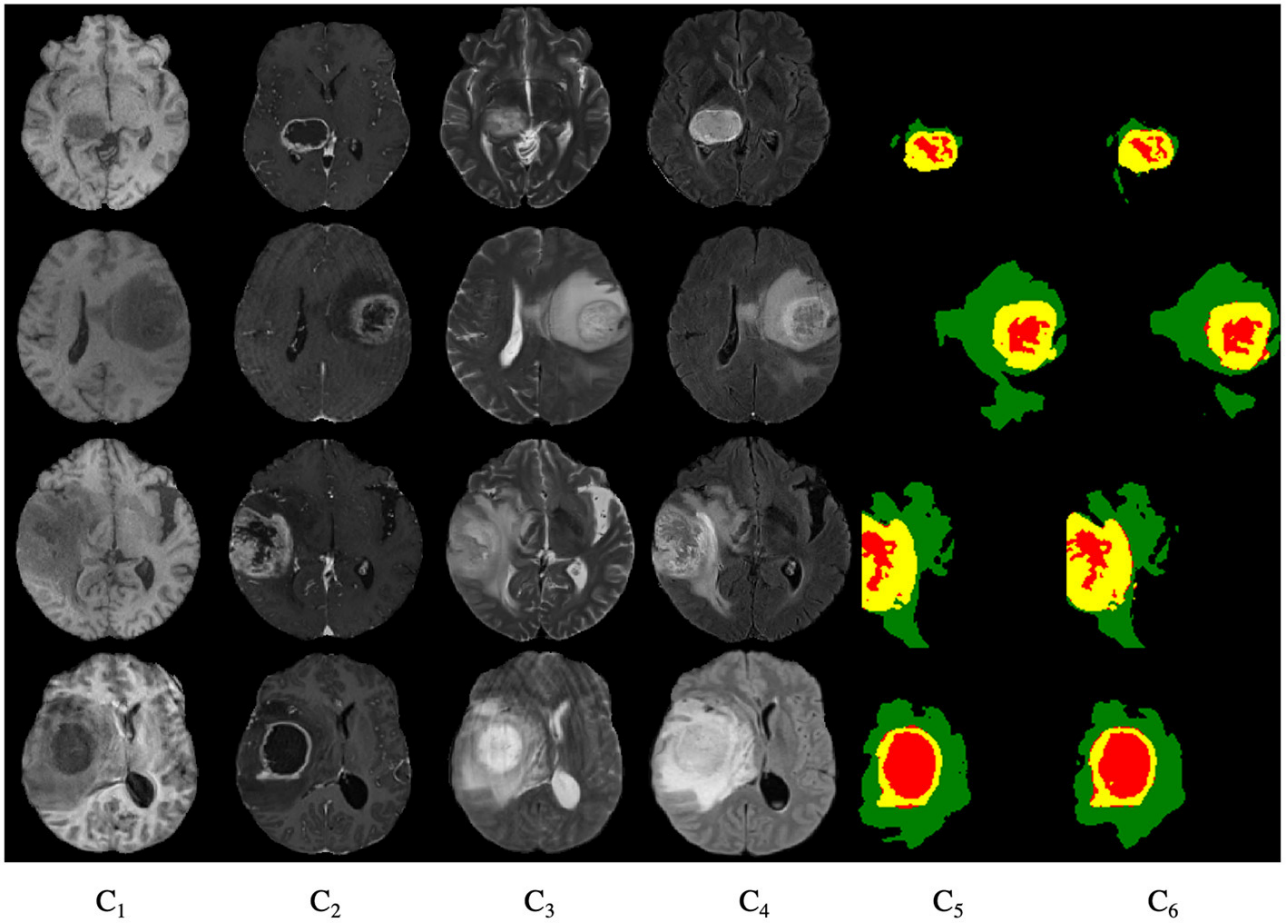
Segmentation results for four cases. *C*_1_ are the images represent one axial MRI slice obtained in T1-weighted, *C*_2_ are the T1c-weighted, *C*_3_ are the T2-weighted, *C*_4_ are the FLAIR-weighted, *C*_5_ are the the standard tumor segmentation results manually labeled by experts, and the *C*_6_ show the segmentation results obtained in this section.

Figure 9 shows the segmentation results for the four cases obtained from the segmentation network of our proposed model. In these graphs, each row represents a real clinical case. From left to right, the images represent one axial MRI slice obtained in T1-weighted, T1c-weighted, T2-weighted, and FLAIR-weighted, the standard tumor segmentation results manually labeled by experts, and the last column shows the segmentation results obtained in this section. The tumor categories are highlighted with different colors: enhancing tumor regions (yellow), peritumor edema regions (green), and necrotic and non-enhancing tumors (red). It can be seen that the tumor size, shape, location, and category differ in the four brain MRIs. As can be seen from the comparison graph, the segmentation network of the Dense-ResUNet model is close to the standard segmentation results of cancer tumors manually labeled by experts, which proves the effectiveness of the Dense-ResUNet model.

## 4. Conclusion

In this article, we design a model based on 2D UNet brain tumor segmentation model and named it as Dense-ResUNet. By combining the features of multi-modal brain tumor MRI images, the attributes of each phase in MRI images were improved. The Dense-ResUNet makes full use of the nested dense convolutional blocks to fill in the gaps of traditional UNet, which capture different levels of features. Then it uses a residual unit to extract pixel information from the image and skip the link to solve the traditional deep CNN network problem. Finally the multi-scale feature maps are fused to obtain the segmentation results. Experiment results show that our proposed method is better than other comparative approaches. The result can prove the effectiveness of our framework and show that the Dense-ResUNet can help extract the fusion of multi-modal of brain tumor images through the image convolutional network, which has clinical research and application value.

## Data Availability Statement

Publicly available datasets were analyzed in this study. This data can be found at: https://aistudio.baidu.com/aistudio/datasetdetail/64660.

## Author Contributions

SC wrote the main manuscript and conducted the experiments. SZ and QL wrote, reviewed, edited, and supervised the manuscript. All authors read and approved the submitted manuscript.

## Conflict of Interest

The authors declare that the research was conducted in the absence of any commercial or financial relationships that could be construed as a potential conflict of interest.

## Publisher's Note

All claims expressed in this article are solely those of the authors and do not necessarily represent those of their affiliated organizations, or those of the publisher, the editors and the reviewers. Any product that may be evaluated in this article, or claim that may be made by its manufacturer, is not guaranteed or endorsed by the publisher.
